# Triglyceride Glucose-Body Mass Index and Risk of Incident Type 2 Diabetes Mellitus in Japanese People With Normal Glycemic Level: A Population-Based Longitudinal Cohort Study

**DOI:** 10.3389/fendo.2022.907973

**Published:** 2022-07-14

**Authors:** Bei Song, Xiaofang Zhao, Tianci Yao, Weilin Lu, Hao Zhang, Ting Liu, Chengyun Liu, Kun Wang

**Affiliations:** Department of Geriatrics, Union Hospital, Tongji Medical College, Huazhong University of Science and Technology, Wuhan, China

**Keywords:** triglyceride glucose-body mass index, incident type 2 diabetes mellitus, cohort study, normal glycemic level, japanese

## Abstract

**Background:**

It has been proved that triglyceride glucose-body mass index (TyG-BMI) is a readily available and clinically significant indicator of insulin resistance (IR). Nevertheless, the association between TyG-BMI and incident Type 2 diabetes mellitus (T2DM) remains uncertain. This study aimed to study the relationship between TyG-BMI and T2DM and explore the predictive characteristics of TyG-BMI.

**Methods:**

Our study was conducted as a longitudinal cohort study. 8,430 men and 7,034 women were enrolled and analyzed. They were both non-diabetic subjects with normal glycemic levels. Follow-up lasted for 13 years, from 1994 to 2016. To make the number of TyG-BMI in each group similar, the subjects were divided into four groups with 3866 subjects in each group.

**Results:**

During the 13-year follow-up period, 373 subjects were diagnosed with incident T2DM. Our multivariate Cox regression analysis revealed that TyG-BMI was an independent predictor of incident T2DM. In addition, our research identified four specific groups, young people (18-44 years old), women, the non-hypertensive population and non-drinkers were at significantly higher risk of developing TyG-BMI-related diabetes (P-interaction< 0.05). The best threshold TyG-BMI for predicting incident T2DM was 197.2987 (area under the curve 0.7738).

**Conclusions:**

Our longitudinal cohort study demonstrated the positive correlation between baseline TyG-BMI and risk of incident T2DM in Japanese with normal glycemic levels, and this risk was significantly higher in the young people, women, the non-hypertensive population and non-drinkers.

## Introduction

Type 2 Diabetes Mellitus (T2DM) is one of the most common metabolic diseases (1). In 2021, More than half a billion adults aged (between 20 and 79) had diabetes mellitus. The total number of people with diabetes is expected to exceed 600 million in 2030 and rise to 783 million in another 15 years. In 2021, diabetes and its complications will cause 6.7 million deaths worldwide (2). The high mortality was mainly due to cardiovascular disease, kidney disease and infection in T2DM patients (3–5). People with impaired glucose tolerance have been shown to be at increased risk for the disease (6). The number of people with T2DM in Japan has been growing. In 2021, Japanese adult diabetes health expenditure will be about 35.6 billion yuan, ranking fifth in the world (2). Therefore, finding the best way to prevent and treat T2DM is crucial. Insulin resistance (IR) is a determining factor in the pathophysiology of T2DM (7). IR has also been a key mechanism in the development and progression of many other metabolic diseases, such as obesity, metabolic syndrome and nonalcoholic fatty liver disease (NAFLD) (8–10). The golden method for IR detection is the hyperinsulinemic-euglycemic clamp (HIEC) technique (11). Nevertheless, this technique requires accurate quantitative infusion equipment and frequent blood glucose measurement, resulting in high cost and high technical requirements (12). A more convenient and cheaper IR measurement is critical for prospective risk assessment, extensive population screening, and treatment monitoring. Triglyceride glucose-body mass index (TyG-BMI) is a new obesity-related parameter that has been developed in recent years, and it’s obtained by multiplying TyG (composed of fasting plasma glucose and triglycerides) with BMI (derived from weight and height) (13–21). These are routine physical examination indicators that are easy to obtain. Recent studies have confirmed that TyG-BMI, as a simple and clinically valuable index, can be used to identify the alternative index of IR at an early stage (21).

As far as we know, there are three observational studies about the relationship between TyG-BMI and Diabetes (14, 17, 22). Among them, the primary outcome of two studies was prediabetes. Compared with the three studies mentioned above, our advantages and differences are as follows: First, our primary outcome of incident T2DM was very clear, while T2DM and type 1 diabetes mellitus (T1DM) were not distinguished in other studies; Second, the follow-up time is longer, our follow-up time is as long as 13 years; Third, the study excluded participants with alcoholic fatty liver disease, viral hepatitis, diagnosed diabetes, diagnosed pre-diabetes and any type of medication were excluded from the study; Fourth, our population is in a different country. Our study population comes from Japan.

Our longitudinal cohort study was a second study based on a large cohort of people with normal glycemic levels in Japan. It aimed to further characterize and analyze the association between baseline TyG-BMI and the incident T2DM.

## Methods and Materials

### Participants and Study Design

**Figure 1:**
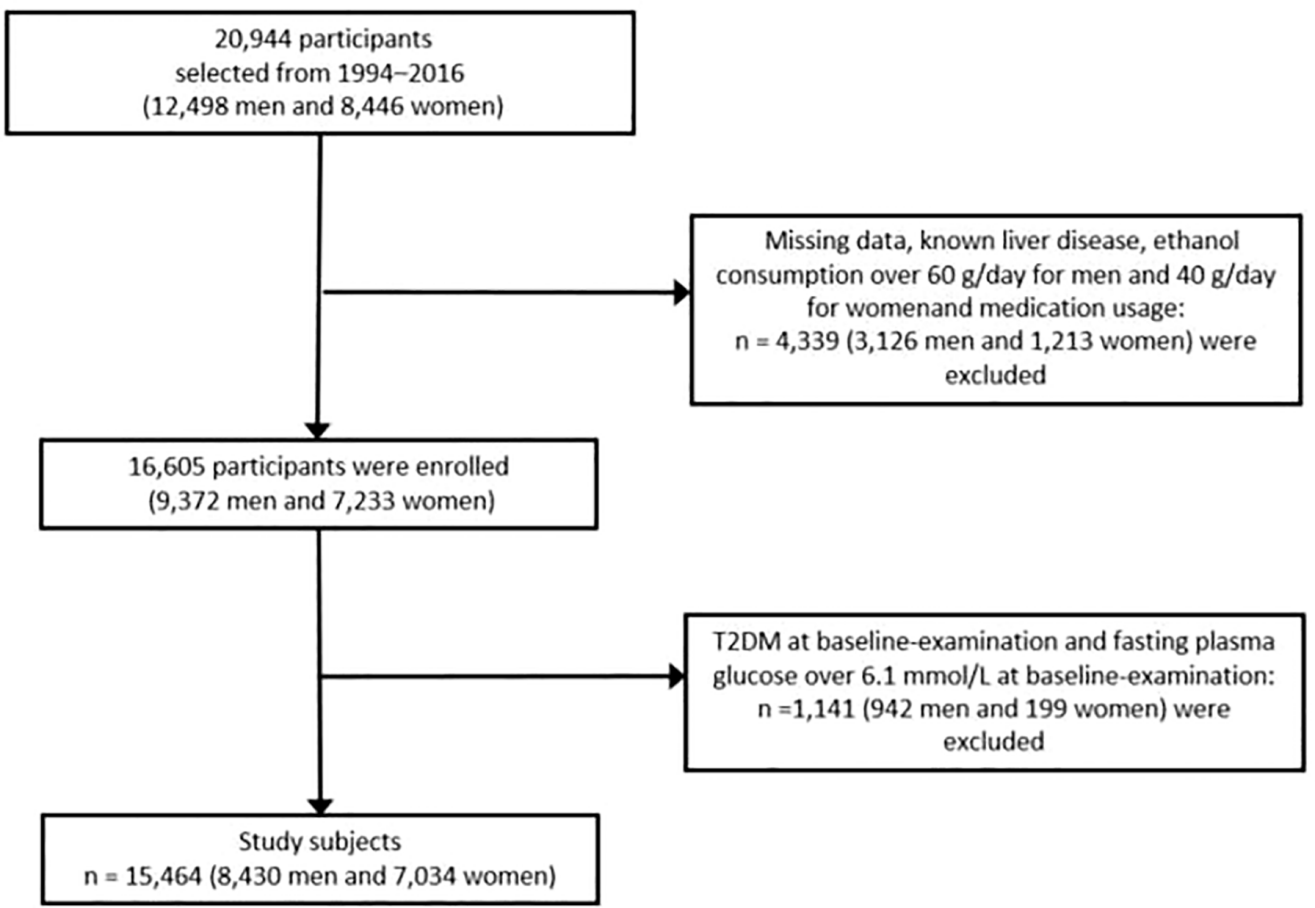
Flow chart showing the exclusion criteria of participants. T2DM, type 2 diabetes mellitus.

We performed this study using data from the NAGALA (NAfld in the Gifu Area, Population-based Longitudinal Analysis) database collected at Murakami Memorial Hospital in Japan. This center was founded in 1994, it can perform More than 8,000 medical examinations per year, and more than half of the patients would have one or two physical check-ups a year (23). From 1994 to 2016, 20944 participants were enrolled in this medical examination program. 12,498 of them are male and 8446 of them are female. Then they excluded people with diabetes at the baseline examination, viral hepatitis, alcoholic fatty liver disease, any drug used in baseline, HbA1c of 6.5% or more, fasting plasma glucose of 7 mmol/L or more and missing covariable data (**Figure 1**). This project has been approved by the Ethics Committee of Murakami Memorial Hospital in Japan at the time of collection, and all participants in this project have signed informed consent to use their physical examination data for research.

### Data Collection and Measurements

They use a standardized self-management behavior questionnaire form to collect data on lifestyle (smoking and alcohol habits and physical activity). The specific definitions of lifestyle (smoking status, drinking status and exercise habits), viral hepatitis, alcoholic fatty liver disease, and incident T2DM can be obtained in the original article described above (23). We described only the following key definitions of our concern. They asked participants how many grams of alcohol they drank per week in the previous month, dividing them into four groups: never, light, moderate and heavy drinkers. They also divided the participants into three groups based on their smoking status: never smoked, never smoked or current smokers. Exercising more than once a week was defined as an exercise habit. The incident T2DM was defined as having an HbA1c of 6.5% or more and fasting plasma glucose of 7 mmol/L (24) or more, or self-reported.

### Definition of Index

The formulas for calculating the indexes: BMI = weight(kg) / height(m)^2^; TyG index = Ln [1/2 fasting plasma glucose (mg/dL × fasting triglycerides (mg/dL)]; TyG – BMI = BMI × TyG index.

### Statistical Analyses

**Figure 2:**
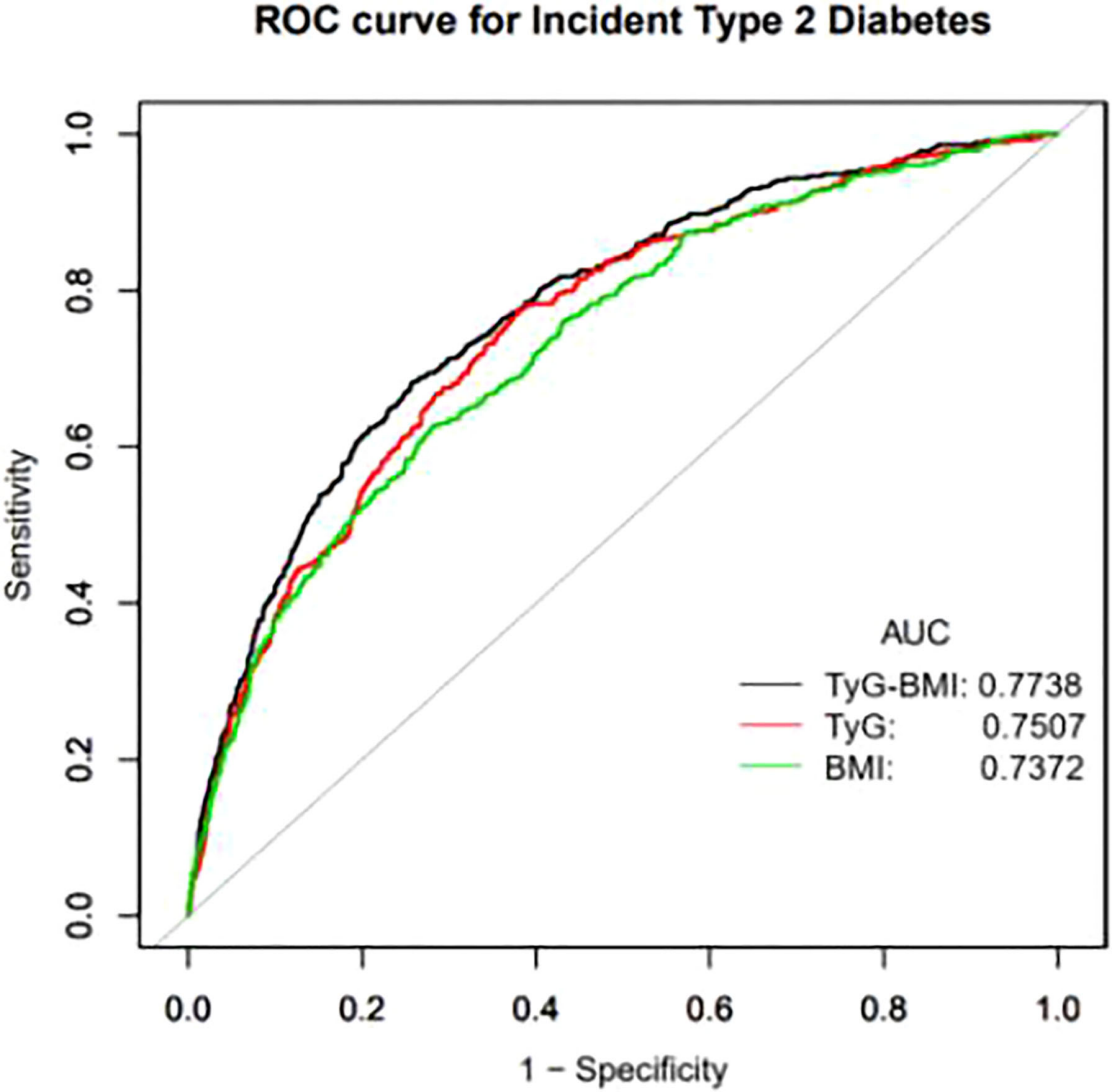
Receiver operating characteristic (ROC) curve analyses to predict incident T2DM. AUC, area under the curve; TyG-BMI, triglyceride glucose-body mass index; TyG, triglyceride-glucose; BMI, body mass index.

The EmpowerStats and R (a free software environment for statistical computing and graphics; http://www.r-project.org) were used for all statistical analyses in our study (25). In **Table 1**, participants are categorized into four groups according to the baseline TyG-BMI quartiles. We used mean ± standard deviation (SD) to represent continuous variable baseline characteristics and frequency (%) to represent categorical variable baseline characteristics. We first tested the normality of the data by Kolmogorov-Smirnov test. In the case of continuous normal distribution, ANOVA or Kruskal–Wallis H tests were used to test the statistical difference between the two groups. If the normality hypothesis of the T-test was not satisfied, we used the Manni-Whitney tests. The Chi-square test was used to analyze the statistical differences between categorical variables. In **Table 2**, Our aim was to assess the association between baseline TYG-BMI levels and the incidence of T2DM using a Multivariate Cox regression analysis. Five models were used for this analysis (1): the primary model without adjustment; (2) the model I adjusted for the most basic variable (baseline sex and age); (3) the model II adjusted for baseline HDL-cholesterol, total cholesterol, triglycerides, drinking, smoking, and exercise habits; (4) model III adjusted for variables in model II and variables with P < 0.001 in univariate analysis (**Supplementary Table 1**); (5) model IV adjusted for all above variables. Finally, in **Table 3**, In this study, the Cox model and likelihood ratio test were used for stratified analysis and it was found that there were significant differences in the relationship between TyG-BMI and newly diagnosed T2DM in different genders, ages, drinking status and blood pressure, with significant interaction. Potential confounders we used in these analyses include baseline age, sex, HDL cholesterol, total cholesterol, triglycerides, habit of exercise, alcohol consumption, smoking status, fatty liver, ALT, AST, GGT, systolic blood pressure (SBP) and diastolic blood pressure (DBP). In **Figure 2** and **Table 4**, receiver operating characteristic (ROC) curve analysis was adopted to calculate the area under the curve (AUC) and best threshold, which presented predictive abilities of the TyG-BMI, TyG index and BMI for incident T2DM risk. A P-value less than 0.05 was considered statistically significant.

**Table 1:** Baseline variables according to the quartile of TyG-BMI.

Variable	TyG-BMI				*P*-value
	Quartile 1 (97.49-153.10)	Quartile 2 (153.11-174.15)	Quartile 3 (174.16-199.26)	Quartile 4 (199.27-421.35)	
N	3866	3866	3866	3866	
Case of Incident T2DM	19 (0.49%)	41 (1.06%)	69 (1.78%)	244 (6.31%)	<0.001
Age, yr	40.74 ± 8.55	43.93 ± 8.84	45.18 ± 8.96	44.98 ± 8.52	<0.001
BMI, kg/m2	18.78 ± 1.35	20.92 ± 1.17	22.77 ± 1.27	26.00 ± 2.51	<0.001
Waist circumference, cm	67.25 ± 4.99	73.15 ± 5.25	78.79 ± 5.17	86.69 ± 6.82	<0.001
ALT, IU/L	14.48 ± 6.68	16.43 ± 7.85	20.28 ± 16.60	28.75 ± 17.92	<0.001
AST, IU/L	16.80 ± 5.68	17.20 ± 6.63	18.35 ± 11.11	21.26 ± 9.32	<0.001
Body Weight, kg	49.37 ± 5.84	56.47 ± 6.59	63.33 ± 7.07	73.39 ± 9.91	<0.001
GGT, IU/L	13.64 ± 9.16	16.31 ± 11.85	21.51 ± 18.59	29.78 ± 24.38	<0.001
HDL-cholesterol, mmol/L	1.72 ± 0.39	1.56 ± 0.37	1.38 ± 0.34	1.18 ± 0.29	<0.001
Total Cholesterol, mmol/L	4.77 ± 0.79	5.05 ± 0.82	5.21 ± 0.83	5.47 ± 0.86	<0.001
Triglycerides, mmol/L	0.46 ± 0.21	0.68 ± 0.28	0.95 ± 0.43	1.56 ± 0.86	<0.001
HbA1c, mmol/mol	32.46 ± 3.27	32.70 ± 3.39	33.04 ± 3.54	33.90 ± 3.69	<0.001
Fasting plasma glucose, mmol/L	4.91 ± 0.38	5.09 ± 0.38	5.24 ± 0.37	5.40 ± 0.36	<0.001
Systolic blood pressure, mmHg	105.65 ± 12.18	111.49 ± 13.25	116.94 ± 13.37	123.91 ± 14.57	<0.001
Diastolic blood pressure, mmHg	65.43 ± 8.47	69.35 ± 9.41	73.31 ± 9.52	78.23 ± 10.02	<0.001
TyG index	7.41 ± 0.45	7.85 ± 0.40	8.20 ± 0.42	8.68 ± 0.50	<0.001
Follow up duration, days	2144.21 ± 1339.26	2216.04 ± 1394.73	2250.28 ± 1385.87	2220.75 ± 1396.58	0.017
Sex					<0.001
Female	2979 (77.06%)	2078 (53.75%)	1188 (30.73%)	789 (20.41%)	
Male	887 (22.94%)	1788 (46.25%)	2678 (69.27%)	3077 (79.59%)	
Fatty liver					<0.001
No	3844 (99.43%)	3726 (96.38%)	3274 (84.69%)	1879 (48.60%)	
Yes	22 (0.57%)	140 (3.62%)	592 (15.31%)	1987 (51.40%)	
Habit of exercise 0/1					<0.001
No	3245 (83.94%)	3111 (80.47%)	3140 (81.22%)	3259 (84.30%)	
Yes	621 (16.06%)	755 (19.53%)	726 (18.78%)	607 (15.70%)	
Alcohol consumption					<0.001
Never	3356 (86.81%)	3002 (77.65%)	2754 (71.24%)	2693 (69.66%)	
Light	288 (7.45%)	449 (11.61%)	521 (13.48%)	500 (12.93%)	
Moderate	179 (4.63%)	314 (8.12%)	419 (10.84%)	448 (11.59%)	
Severe	43 (1.11%)	101 (2.61%)	172 (4.45%)	225 (5.82%)	
Smoking status					<0.001
Never	2987 (77.26%)	2461 (63.66%)	1924 (49.77%)	1659 (42.91%)	
Past	391 (10.11%)	651 (16.84%)	923 (23.87%)	987 (25.53%)	
Current	488 (12.62%)	754 (19.50%)	1019 (26.36%)	1220 (31.56%)	

Continuous variables are presented as mean ± S.D. or as median (Q1-Q4). Categorical data are presented as frequencies (percentages).

Q, quartile; ALT, alanine aminotransferase; AST, aspartate aminotransferase; GGT, gamma-glutamyl transpeptidase; DBP, diastolic blood pressure; SBP, systolic blood pressure; HDL-cholesterol, high density lipoprotein-cholesterol; HbA1c, Hemoglobin A1c; TyG-BMI, triglyceride glucose-body mass index; TyG, triglyceride-glucose; BMI, body mass index.

**Table 2:** Associations of baseline TyG-BMI with incident T2DM.

	HR (95%CI)					
	Multivariable Analysis (continuous)	TyG-BMI quartile				*P*-trend
		Q1	Q2	Q3	Q4	
Crude Model	1.024 (1.022, 1.027)	Ref	2.034 (1.181, 3.505)	3.348 (2.015, 5.564)	12.031 (7.543, 19.189)	<0.00001
Model I	1.025 (1.023, 1.028)	Ref	1.700 (0.983, 2.941)	2.516 (1.492, 4.244)	8.952 (5.492, 14.591)	<0.00001
Model II	1.020 (1.017, 1.023)	Ref	1.629 (0.940, 2.824)	2.074 (1.218, 3.530)	5.397 (3.178, 9.165)	<0.00001
Model III	1.013 (1.009, 1.017)	Ref	1.520 (0.875, 2.641)	1.490 (0.863, 2.572)	2.376 (1.336, 4.225)	0.00138
Model IV	1.015 (1.011, 1.019)	Ref	1.419 (0.816, 2.470)	1.400 (0.809, 2.421)	2.280 (1.285, 4.045)	0.00135

Crude model adjusted for None.

Model I adjusted for baseline age and sex.

Model II adjusted for baseline HDL-cholesterol, total cholesterol, triglycerides, alcohol consumption, smoking, habits of exercise.

Model III adjusted for baseline SBP, DBP, fatty liver, ALT, AST, GGT, HDL-cholesterol, total cholesterol, triglycerides, alcohol consumption, smoking, habits of exercise.

Model IV adjusted for baseline age, sex, HDL-cholesterol, SBP, DBP, total cholesterol, triglycerides, alcohol consumption, smoking status, habits of exercise, fatty liver, ALT, AST, GGT.

HR, hazard ratio; CI, confidence intervals; TyG-BMI, triglyceride glucose-body mass index; TyG, triglyceride-glucose; BMI, body mass index.

**Table 3:** Stratified association between TyG-BMI and diabetes by age, sex, alcohol consumption, smoking status, SBP and DBP.

Subgroup	ParticipantsN	unadjusted H.R. (95%CI) *P*-value	adjusted HR (95%CI) *P*-value	*P* for interaction
Age (years)				0.0477
18-44	8,872	1.027 (1.024, 1.029) <0.0001	1.021 (1.017, 1.025) <0.0001	
45-79	6,592	1.022 (1.018, 1.026) <0.0001	1.016 (1.011, 1.021) <0.0001	
Sex				0.0123
Male	8,430	1.022 (1.019, 1.024) <0.0001	1.013 (1.008, 1.017) <0.0001	
Female	7,034	1.030 (1.026, 1.035) <0.0001	1.020 (1.014, 1.026) <0.0001	
Alcohol consumption				0.0457
Never	11,805	1.025 (1.023, 1.028) <0.0001	1.020 (1.016, 1.023) <0.0001	
Light	1,758	1.025 (1.017, 1.032) <0.0001	1.017 (1.009, 1.026) <0.0001	
Moderate	1,360	1.019 (1.009, 1.028) 0.0002	1.012 (1.001, 1.022) 0.0101	
Severe	541	1.010 (0.999, 1.021) 0.0695	1.004 (0.992, 1.016) 0.2547	
Smoking status				0.0857
Never	9,031	1.025 (1.022, 1.028) <0.0001	1.017 (1.012, 1.022) <0.0001	
Past	2,952	1.020 (1.014, 1.027) <0.0001	1.008 (1.000, 1.016) <0.0001	
Current	3,481	1.023 (1.019, 1.026) <0.0001	1.014 (1.009, 1.019) <0.0001	
SBP (mmHg)				0.0251
≤140	14,704	1.027 (1.024, 1.030) <0.0001	1.023 (1.019, 1.027) <0.0001	
>140	760	1.015 (1.009, 1.021) <0.0001	1.015 (1.008, 1.021) <0.0001	
DBP (mmHg)				0.0296
≤90	14,753	1.027 (1.024, 1.029) <0.0001	1.022 (1.018, 1.026) <0.0001	
>90	711	1.015 (1.009, 1.021) <0.0001	1.014 (1.007, 1.021) <0.0001	

Adjusted for baseline age, sex, HDL-cholesterol, SBP, DBP, total cholesterol, triglycerides, alcohol consumption, smoking status, habits of exercise, ALT, AST, GGT.

DBP, diastolic blood pressure; SBP, systolic blood pressure; TyG-BMI, triglyceride glucose-body mass index.

**Table 4:** AUC with the 95% CI of TyG-BMI, TyG index and BMI for predicting incident T2DM.

Test	ROC area (AUC)	95%CI low	95%CI up	Best threshold	Specificity	Sensitivity
TyG-BMI	0.7738	0.7498	0.7979	197.2987	0.7416	0.6836
TyG index	0.7505	0.7255	0.7754	8.1960	0.6198	0.7748
BMI	0.7327	0.7068	0.7585	23.5285	0.7182	0.6273

AUC, area under the curve; CI, confidence interval; TyG-BMI, triglyceride glucose-body mass index; TyG, triglyceride-glucose index; BMI, body mass index.

## Results

### Study Population Description Based on TyG-BMI Quartiles

After excluding people with diabetes at the baseline examination, viral hepatitis, alcoholic fatty liver disease, any drug used in the baseline, our cohort study eventually included 15464 participants (8430 men and 7034 women) (**Figure 1**). The mean age of our cohort was 43.71 ± 8.90 years. After the mean follow up duration of 2207.82 ± 1379.72 days, incident T2DM occurred in 373 (2.41%) participants (**Supplementary Table 2**). It was clear that most baseline variables were statistically significantly differences between the four groups. Compared with participants in quartile 1 which had the lowest BMI, participants in quartile 4 were older, heavier, more likely to be male, had bigger waist circumference, higher total cholesterol, higher triglycerides, HbA1c, AST, ALT, GGT, blood pressures and fasting plasma glucose, more had fatty liver and incident T2DM, more smokers, more drinker. However, participants of quartile 4 were lower HDL-cholesterol levels, fewer regular exercisers, non-smokers and non-drinkers (P<0.001) (**Table 1**).

### The Association Between Incident T2DM and Baseline TyG-BMI

This study evaluated the relationship between TyG-BMI and incident T2DM by Cox regression model. In order to verify the stability of the relationship under different conditions, four models were established (**Table 2**). First of all, the model I only adjusted for baseline age and sex. The results of regression analysis by this model showed that TyG-BMI was significantly positively correlated with the risk of incident T2DM (P<0.001). Meanwhile, it could be seen from the results of this model that the risk of diabetes corresponding to TyG-BMI was significantly increased in quartile 4 compared with quartile 1. Model II adjusted for confounding factors about blood lipids and lifestyle, such as baseline HDL-cholesterol, total cholesterol, triglycerides, alcohol consumption, smoking, habits of exercise. The results of this model suggest that TyG-BMI is still positively and significantly associated with the risk of diabetes, but it is weaker than the model I (P<0.001). Model III adjusted for variables in model II and variables that were significantly associated with the risk of developing incident T2DM in univariate analysis (P<0.001). However, nonlinear relationships begin to emerge between TyG-BMI and incident T2DM in this model, but the general trend remains the same (p<0.05). Finally, we adjusted for almost all variables above in model IV and the trend remains unchanged and elevated TyG-BMI remains a risk factor for incident T2DM (p<0.05). In a word, we conclude that TyG-BMI is an independent risk factor for incident T2DM. And as a continuous variable, the relationship between baseline TyG-BMI and incident T2DM was nearly positively linear.

### Subgroup Analysis and Interaction Test

**Table 3** evaluates the interaction of age, sex, alcohol consumption, smoking status and blood pressure, with TyG-BMI and incident T2DM. Within these stratifications, young adults, women, non-drinkers, and baseline non-hypertensive individuals were at a significantly higher risk of developing TyG-BMI-related Diabetes than their counterparts (P-interaction< 0.05).

### Predictive Value of TyG-BMI in Incident T2DM

A ROC curve drawn by our researchers, which aims to more clearly demonstrate the predictive value of TyG-BMI, TyG index and BMI on the incidence of T2DM. The area under the curve (AUC) of TyG-BMI was 0.7738 (0.7498, 0.7979), which was higher than the AUC of TyG and BMI. The AUC of TyG index 0.7505 (0.7255,0.7754) and the AUC of BMI was 0.7327 (0.7068, 0.7585) (**Figure 2**). The best threshold of TyG-BMI that we focused on was 197.2987, with a sensitivity of 74.16% and specificity of 68.36% (**Table 4**).

## Discussion

After reviewing numerous studies, we believed that this was the first retrospective cohort study in a Japanese population to describe the association between TyG-BMI and the risk of T2DM in subjects with normal glycemic levels. In this cohort study, we found that TyG-BMI was always stable in different models and was independently positively associated with the incidence of T2DM. Meanwhile, we found some particular phenomena in the subgroup analysis. There are significant differences between TyG-BMI and the risk of incident T2DM among people of different ages, genders, blood pressure and alcohol consumption. Among them, the independent correlation between TyG-BMI and the incidence of T2DM was more evident in young people (18-44 years old), women, non-hypertensive people and non-drinkers. It is not clear why TyG-BMI is associated with a significantly increased risk of Diabetes in these populations. However, the following points need to be noted: (1) With the rapid development of society, young people develop more and more unhealthy living habits due to the increasing pressure and entertainment life, which affects their metabolism (26–28). (2) Body composition and metabolism vary significantly between men and women, with men generally having higher muscle mass and faster metabolism (29, 30). (3) Alcohol consumption affects the liver, lipid metabolism and gut microbiome composition, even if it is moderate (31) (4). Long-term high blood pressure can also aggravate Diabetes. For example, high blood pressure can cause systemic arteriosclerosis, which affects the function of the islet (32, 33).

### Comparisons With Other Studies and What Does the Current Work add to the Existing Knowledge

TyG, a product calculated from triglycerides and glucose, is a readily available measure of insulin sensitivity (34). New research showed that the TyG index may be an important prognostic indicator of prediabetes patients and can independently predict new cardiovascular and cerebrovascular adverse events (35). Some studies have also proved that with the increase of TyG index, the risk of Diabetes also significantly increased and it was a risk markers of insulin resistance (36–38). BMI was known to be used to assess the risk of obesity and metabolic diseases and is the most readily available indicator calculated by height and weight (39). An elevated BMI increases the risk of Diabetes (40). TyG-BMI was the product of BMI multiplied by the TyG index and a new obesity-related parameter that has been developed in recent years. Compared with TyG, BMI, TG and fasting blood glucose, TyG-BMI has an advantage in determining NAFLD risk in nonobese patients (20).In 2020, the results showed that TyG-BMI was positively correlated with the incidence of NAFLD in the normal lipid levels and nonobese subgroups of the Chinese population (18). In August 2021, Khamseh et al. proved that TyG-BMI could reliably predict liver fibrosis in overweight or obese people without Diabetes (16). In November of the same year, Jiang et al. found in a cohort study that a higher TyG-BMI would significantly increase the risk of prediabetes in the population, especially in women, nonobese people and people under 50 years old (17). Apart from that, Early studies have found that TyG-BMI, which consists of fasting blood glucose, triglyceride and obesity status, can more efficiently predict IR than TyG index, BMI and other obesity indexes (21). In conclusion, these studies suggest that TyG-BMI has a predictive value in metabolic diseases. At the same time, a study also found a strong relationship between TyG-BMI and ischemic stroke. Their results also suggest the potential use of TyG-BMI in improving risk stratification for ischemic stroke (19). However, studies examining the relationship between emerging Diabetes and TyG-BMI are limited, with only one relevant study. In the Chinese population, Wang et al. found a causal relationship between TyG-BMI and the incidence of diabetes and this independent relationship was more pronounced in younger people and nonobese individuals (14). This study had the following limitations (1): the diagnosis of diabetes in their primary outcome did not distinguish between T2DM and T1DM; they only found a relationship between TyG-BMI and new-onset diabetes mellitus; (2) In their study, the diagnosis of diabetes was defined as fasting blood glucose greater than 7.0 mmol/L or self-reported, while ignoring the measurement of HbA1C, which could lead to a false negative for the incidence of Diabetes mellitus; (3) Participants in their study were followed for only about seven years, which resulted in a lower incidence of endpoint events and distorted results. Correspondingly, our study makes up for most of the above limitations of the Chinese data cohort study: (1) The primary outcome collected in our cohort was incident T2DM, which was clear and unambiguous; (2) We defined the diagnosis of T2DM as self-reported, HbA1C of at least 6.5% or fasting blood glucose of at least 7.0 mmol/L, which is more accurate; (3) we collected data for a more extended follow-up period, up to 13 years. In addition, our study population was not only in different countries. And the results of our subgroup analysis of interactions are also different. Their subgroup analysis results show that participants who were young people, middle-aged people and people with a BMI of less than 24 kg/m2 have a higher risk of Diabetes mellitus associated with TyG-BMI. In contrast, our subgroup analysis results show that young people (18-44 years old), women, the non-hypertensive population and non-drinkers had a higher risk of developing diabetes mellitus associated with TyG-BMI.

### Strength and Limitations of This Study

However, our study certainly had some limitations. First of all, our current study was based only on the Japanese population, making it uncertain whether the results would apply to people in other countries. This means that further research on different ethnic groups is needed. In addition, although our diagnosis of T2DM takes into account HbA1c and fasting glucose, the prevalence of new-onset diabetes may be more accurate if oral glucose tolerance tests are added. Although the population size of our database is large then other studies, it is still small compared with that of the Chinese database, but it is understandable because there is a large gap between the total population. Despite these limitations, the study has several advantages. First of all, the study was conducted in people with normal glycemic levels, excluding diabetes and pre-diabetes (HbA1C≥6.5% or fasting blood glucose≥7.0 mmol/L), which is different from previous studies, so our results may be more applicable to people with normal blood sugar levels. Besides, compared to prior studies, our population is different. The original data used in our study is from Japanese physical examination data. Due to the different development rates among different countries and significant differences among individuals, it has more targeted guidance significance for the Japanese. What’s more, compared with previous studies, our analysis adjusted for more confounding factors, making the results more reliable. Finally, our study had a longer follow-up period than other studies. Our follow-up lasted for 13 years.

## Conclusions

Overall, we found that TyG-BMI was independently positively associated with the incident T2DM after adjusting a large number of confounders. This independent relationship was significantly higher in young people (18-44 years old), women, the non-hypertensive population and non-drinkers. And our findings maybe provide evidence for the predictive value of TyG-BMI in preventing incident T2DM.

## Data Availability Statement

Publicly available datasets were analyzed in this study. This data can be found here: https://datadryad.org/stash/dataset/doi:10.5061%2Fdryad.8q0p192.

## Ethics Statement

The studies involving human participants were reviewed and approved by Murakami Memorial Hospital Ethics Committee. The patients/participants provided their written informed consent to participate in this study.

## Author Contributions

BS contributed to design of the study and writing most first draft. BS and XZ organized the database and responded to the editor and reviewers together. TY and HZ performed the statistical analysis. TL and WL participated in the critical modification of important knowledge contents. CL and KW initiated and helped design the study. And they also ensured the accuracy or completeness of all questions in the study. All authors contributed to the article and approved the submitted version.

## Funding

All costs of this study were supported by National Natural Science Foundation of China (NSFC) project 81974222.

## Conflict of Interest

The authors declare that the research was conducted in the absence of any commercial or financial relationships that could be construed as a potential conflict of interest.

## Publisher’s Note

All claims expressed in this article are solely those of the authors and do not necessarily represent those of their affiliated organizations, or those of the publisher, the editors and the reviewers. Any product that may be evaluated in this article, or claim that may be made by its manufacturer, is not guaranteed or endorsed by the publisher.
