# Supplementary material for: Triglyceride Glucose-Body Mass Index and Risk of Incident Type 2 Diabetes Mellitus in Japanese People With Normal Glycemic Level: A Population-Based Longitudinal Cohort Study
Source: Front Endocrinol (Lausanne). 2022 Jul 14;13:907973. doi: 10.3389/fendo.2022.907973 (PMC9336540; doi:10.3389/fendo.2022.907973)
Supplement: Supplementary file 1 [file DataSheet_1.docx]

Supplementary Material

# Supplementary Data

none

# Supplementary Tables

**Supplementary Table 1**

**Description of baseline variables.**

| Variable | Mean+SD |
| --- | --- |
| Age, yr | 43.71 ± 8.90 |
| BMI, kg/m2 | 22.12 ± 3.13 |
| Waist circumference, cm | 76.47 ± 9.11 |
| ALT, IU/L | 19.99 ± 14.34 |
| AST, IU/L | 18.40 ± 8.64 |
| Body Weight, kg | 60.64 ± 11.62 |
| GGT, IU/L | 20.31 ± 18.14 |
| HDL-cholesterol, mmol/L | 1.46 ± 0.40 |
| Total Cholesterol, mmol/L | 5.13 ± 0.86 |
| Triglycerides, mmol/L | 0.91 ± 0.66 |
| HbA1c, mmol/mol | 33.03 ± 3.52 |
| Fasting plasma glucose, mmol/L | 5.16 ± 0.41 |
| SBP, mmHg | 114.50 ± 14.97 |
| DBP, mmHg | 71.58 ± 10.50 |
| TyG | 8.03 ± 0.65 |
| TyG-BMI | 178.60 ± 34.54 |
| Follow up duration, days | 2207.82 ± 1379.72 |
|  | N (%) |
| Sex |  |
| Female | 7034 (45.49%) |
| Male | 8430 (54.51%) |
| Fatty liver |  |
| No | 12723 (82.27%) |
| Yes | 2741 (17.73%) |
| Habit of exercise 0/1 |  |
| No | 12755 (82.48%) |
| Yes | 2709 (17.52%) |
| Alcohol consumption |  |
| Never | 11805 (76.34%) |
| Light | 1758 (11.37%) |
| Moderate | 1360 (8.79%) |
| Severe | 541 (3.50%) |
| Smoking status |  |
| Never | 9031 (58.40%) |
| Past | 2952 (19.09%) |
| Current | 3481 (22.51%) |
| Incident T2DM |  |
| No | 15091 (97.59%) |
| Yes | 373 (2.41% |

Abbreviation: ALT, alanine aminotransferase; AST, aspartate aminotransferase; GGT, gamma-glutamyl transpeptidase; DBP, diastolic blood pressure; SBP, systolic blood pressure; HDL-cholesterol, high density lipoprotein-cholesterol; HbA1c, Hemoglobin A1c; TyG-BMI, triglyceride glucose-body mass index; TyG, triglyceride-glucose; BMI, body mass index.

**Supplementary Table 2**

Univariate Cox proportional hazard models between baseline variables and incident T2DM.

| Statistics | Incident T2DM |  | P-value |
| --- | --- | --- | --- |
| Sex |  |  |  |
| Female | 7034 (45.49%) | 1 |  |
| Male | 8430 (54.51%) | 2.52 (1.98, 3.21) | <0.0001 |
| Age, yr | 43.71 ± 8.90 | 1.06 (1.04, 1.07) | <0.0001 |
| Fatty liver |  |  |  |
| No | 12723 (82.27%) | 1 |  |
| Yes | 2741 (17.73%) | 7.02 (5.70, 8.63) | <0.0001 |
| BMI, kg/m2 | 22.12 ± 3.13 | 1.24 (1.22, 1.27) | <0.0001 |
| Waist circumference, cm | 76.47 ± 9.11 | 1.09 (1.08, 1.10) | <0.0001 |
| TyG | 8.03 ± 0.65 | 3.76 (3.22, 4.38) | <0.0001 |
| TyG-BMI | 178.60 ± 34.54 | 1.02 (1.02, 1.03) | <0.0001 |
| ALT, IU/L | 19.99 ± 14.34 | 1.01 (1.01, 1.01) | <0.0001 |
| AST, IU/L | 18.40 ± 8.64 | 1.01 (1.01, 1.01) | <0.0001 |
| Body Weight, kg | 60.64 ± 11.62 | 1.06 (1.05, 1.06) | <0.0001 |
| Habit of exercise 0/1 |  |  |  |
| No | 12755 (82.48%) | 1 |  |
| Yes | 2709 (17.52%) | 0.76 (0.56, 1.02) | 0.0641 |
| GGT, IU/L | 20.31 ± 18.14 | 1.01 (1.01, 1.01) | <0.0001 |
| HDL-cholesterol, mmol/L | 1.46 ± 0.40 | 0.15 (0.11, 0.20) | <0.0001 |
| Total Cholesterol, mmol/L | 5.13 ± 0.86 | 1.49 (1.34, 1.66) | <0.0001 |
| Triglycerides, mmol/L | 0.91 ± 0.66 | 1.80 (1.68, 1.92) | <0.0001 |
| HbA1c, mmol/mol | 33.03 ± 3.52 | 1.44 (1.40, 1.48) | <0.0001 |
| Alcohol consumption |  |  |  |
| Never | 11805 (76.34%) | 1 |  |
| Light | 1758 (11.37%) | 0.90 (0.65, 1.26) | 0.5508 |
| Moderate | 1360 (8.79%) | 1.15 (0.82, 1.62) | 0.4240 |
| Severe | 541 (3.50%) | 2.24 (1.54, 3.27) | <0.0001 |
| Smoking status |  |  |  |
| Never | 9031 (58.40%) | 1 |  |
| Past | 2952 (19.09%) | 1.65 (1.26, 2.18) | 0.0004 |
| Current | 3481 (22.51%) | 2.58 (2.06, 3.24) | <0.0001 |
| Fasting plasma glucose, mmol/L | 5.16 ± 0.41 | 25.37 (18.71, 34.42) | <0.0001 |
| Systolic blood pressure, mmHg | 114.50 ± 14.97 | 1.03 (1.03, 1.04) | <0.0001 |
| Diastolic blood pressure, mmHg | 71.58 ± 10.50 | 1.05 (1.04, 1.06) | <0.0001 |

Abbreviation: ALT, alanine aminotransferase; AST, aspartate aminotransferase; GGT, gamma-glutamyl transpeptidase; DBP, diastolic blood pressure; SBP, systolic blood pressure; HDL-cholesterol, high density lipoprotein-cholesterol; HbA1c, Hemoglobin A1c; TyG-BMI, triglyceride glucose-body mass index; TyG, triglyceride-glucose; BMI, body mass index.

## Supplementary Figures


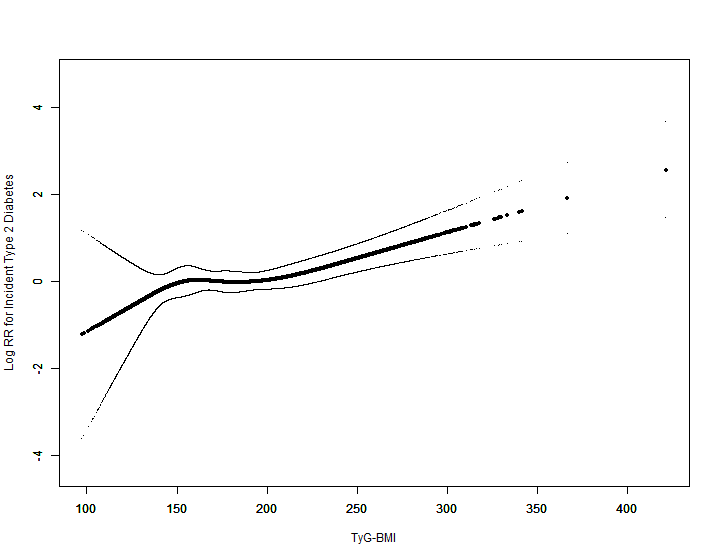


**Supplementary Figure 1.** A smooth curve of the relationship between the TyG-BMI and incident T2DM
